# Cross-sectional study of cholinergic urticaria subtypes and bronchial hyperresponsiveness

**DOI:** 10.1038/s41598-022-22655-6

**Published:** 2022-10-27

**Authors:** Naoko Katsurada, Tatsuya Nagano, Masatsugu Yamamoto, Tatsunori Kiriu, Ryota Dokuni, Hiroshi Kamiryo, Ai Yoshioka, Atsushi Fukunaga, Chikako Nishigori, Yoshihiro Nishimura, Kazuyuki Kobayashi

**Affiliations:** 1 Division of Respiratory Medicine, Department of Internal Medicine, Kobe University Graduate School of Medicine, 7-5-1 Kusunoki-Cho, Chuo-Ku, Kobe, 650-0017 Japan; 2 Division of Dermatology, Department of Internal Related, Kobe University Graduate School of Medicine, Kobe, Japan

**Keywords:** Skin diseases, Asthma

## Abstract

Cholinergic urticaria (CholU) is classified into several subtypes: (1) conventional sweat allergy-type CholU (conventional SAT-CholU), (2) CholU with palpebral angioedema (CholU-PA), 3) CholU with acquired anhidrosis and/or hypohidrosis (CholU-Anhd); 1) and 2) include SAT based on pathogenesis. There have been no studies on differences in the prevalence of bronchial asthma among the subtypes. We analyzed bronchial responsiveness using the methacholine dose indicator D_min_, respiratory symptoms, and exhaled nitric oxide (FeNO). Median log10 D_min_ (interquartile range) of patients with conventional SAT-CholU (n = 11), CholU-PA (n = 11), and CholU-Anhd (n = 11) was 0.381 (− 0.829, 1.079), 0.717 (0.249, 0.787), and 1.318 (0.121, 1.699), respectively (p = 0.516). Respiratory symptoms were less frequently observed in CholU-Anhd than in conventional SAT-CholU or CholU-PA. FeNO of patients with conventional SAT-CholU, CholU-PA, and CholU-Anhd was 23 (18.5, 65.0), 39 (32.0, 59.5), and 25 (19.0, 33.0) ppb, respectively (p = 0.237). Nine% of conventional SAT-CholU patients and more than half of CholU-PA patients required treatment for asthma. Log D_min_ tended to be lower in patients with SAT-CholU than in those with CholU-Anhd. CholU-PA might be associated with asthma.

## Introduction

Cholinergic urticaria (CholU) is characterized by pruritic wheals with surrounding erythema triggered by an increase in core body temperature that is caused by exercise, high environmental temperature, or emotional stress^1^. Patients’ complaints of stinging, tingling pain, or itching usually resolve within 1 h. Respiratory and other severe symptoms such as angioedema and anaphylaxis have been reported to accompany CholU^2,3^, which is most common in young adults with an estimated prevalence of 4–11%^4,5^. Although the precise underlying mechanism is unclear, histamine, cholinergic agents, sweat allergy, serum factors, poral occlusion, and anhidrosis are associated with symptom onset. CholU can be classified into the following several subtypes based on dermatologic characteristics: (1) conventional sweat allergy type (conventional SAT-CholU), (2) CholU with palpebral angioedema (CholU-PA), (3) CholU with acquired anhidrosis and/or hypohidrosis (CholU-Anhd), and other rare subtypes such as follicular-type CholU with a positive autologous serum skin test result^6^. Conventional SAT-CholU is associated with sweat allergy; the same is true of CholU-PA, which has more severe symptoms than conventional SAT-CholU and is accompanied by palpebral angioedema around the eyelids and is strongly associated with atopic diseases such as atopic dermatitis, asthma, and allergic rhinitis^7^. Almost all patients with CholU-PA are female. As conventional SAT-CholU and CholU-PA are both associated with type I allergy to sweat and atopic diseases, they can be grouped as SAT. CholU-Anhd is characterized by acquired generalized hypohidrosis or anhidrosis without sweat allergy. In contrast to conventional SAT-CholU and CholU-PA, CholU-Anhd is not associated with atopic diseases. In CholU-Anhd patients, reduction of acetylcholine receptor M3 on the epithelial cells of sweat glands and Ach-degrading enzyme acetylcholine esterase are seen in hypohidrotic area, and it is thought that the overflow of acetylcholine leaks into mast cells and causes wheal^8,9^.

A previous study showed that 13% of patients with CholU have asthma^10^. And another study reported that 40% of patients with CholU-PA had current or a history of asthma^7^, which is characterized by chronic airway inflammation and bronchial hyperresponsiveness^11^. In a previous study, bronchial hyperresponsiveness was more frequently observed in CholU patients without history of asthma (43%) than in chronic urticaria patients and healthy adults (7%)^12^. However, there have been no studies on differences in the prevalence of asthma among CholU subtypes. This was investigated in the present study by evaluating bronchial responsiveness in each subtype of CholU. We hypothesized that a lack of bronchial hyperresponsiveness would be observed in CholU-Anhd based on a pathogenesis that does not include allergic reaction.

## Results

**Figure 1:**
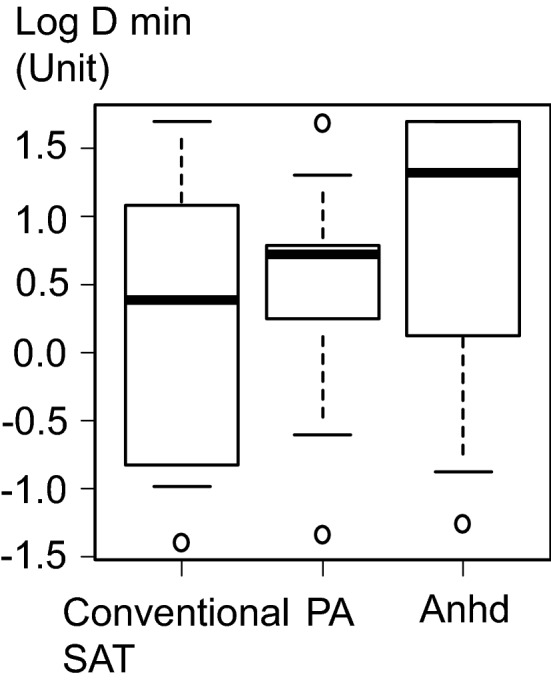
Log10-transformed D_min_ of each CholU subtype. *Anhd* acquired anhidrosis and/or hypohidrosis, *D*_*min*_ cumulative dose of inhaled methacholine when respiratory resistance began to increase, *PA* palpebral angioedema, *SAT* sweat allergy type, *Anhd* acquired anhidrosis and/or hypohidrosis.

The baseline characteristics of the patients are shown in Table 1. A total of 33 patients were enrolled including 11 with conventional SAT-CholU, 11 with CholU-PA, and 11 with CholU-Anhd; thus, 22 patients had SAT. According to a previous report, the amount of inhaled methacholine accumulated before Rrs began to increase (Dmin) was determined as 2 U (1 power of 2) of methacholine 0.049 mg/ml solution inhaled for 1 min. The mean ± standard deviation of log2-transformed Dmin was 4.30 ± 1.80 in 133 asthma patients and 9.5 ± 1.80 in 85 healthy subjects^13^. In pairwise comparisons among the 3 groups, if the difference between the means of the log2-transformed Dmin between the 2 groups is 2.6 and the standard deviation is 1.8, the level of significance is 1.7 (= 5/3) %, the detection rate is 80%, and the number of cases needed in each group is 11 (for a total of 33 cases). There were no patients with follicular-type CholU. All CholU-PA patients were female and all CholU-Anhd patients were male. Five of the 11 patients (45.5%) with CholU-PA had a history of asthma. All the history of asthma preceded the onset of CholU by 2–27 years. No patients were being treated for asthma at the time of enrollment in the study. There was no patients using biologics. Median IgE level (interquartile range) was significantly lower in patients with CholU-Anhd (60.3 [35.2, 127.3] IU/ml) than in those with conventional SAT-CholU (743.7 [539.7, 1580.4] IU/ml) or CholU-PA (360.6 [218.2, 1197.7] IU/ml) (p < 0.001). There were no differences in baseline respiratory resistance (Rrs) before inhalation of methacholine; median baseline Rrs (interquartile range) of patients with conventional SAT-CholU, CholU-PA, and CholU-Anhd was 3.8 (2.45, 4.53), 3.6 (3.25, 4.50), and 3.9 (3.15, 4.00), respectively (p = 0.869). Figure 1 and Supplementary Fig. S1 show log10- and log2-transformed D_min_, respectively, for each CholU subtype. There were no significant differences among the 3 subtypes; median log D_min_ (interquartile range) of patients with conventional SAT-CholU, CholU-PA, and CholU-Anhd was 0.381 (− 0.829, 1.079), 0.717 (0.249, 0.787), and 1.318 (0.121, 1.699), respectively (p = 0.516). The proportion of patients with Rrs that was not increased at 50 U was 18.2% (2/11) for conventional SAT-CholU, 0% (0/11) for CholU-PA, and 36.4% (4/11) for CholU-Anhd (p = 0.127). There was no difference D_min_ between CholU-PA with a history of asthma and those without a history of asthma. Median log D_min_ (interquartile range) of CholU-PA patients with history of asthma and without asthma was 0.750 (0.471, 0.754) and 0.676 (0.179, 0.795), respectively (p = 0.931).

**Table 1:** Patient characteristics for each subtype of CholIU.

Characteristic	Conventional sweat allergy type (n = 11)	CholU with palpebral angioedema (n = 11)	CholU with acquired anhidrosis (n = 11)	p value
Age, years	27 (20, 60)	33 (17, 49)	36 (16, 68)	0.933
Sex, male	8 (72.7)	0 (0.0)	11 (100.0)	< 0.001
History of asthma	2 (18.2)	5 (45.5)	1 (9.1)	0.202
Atopic dermatitis	6 (54.5)	7 (70.0)	0 (0.0)	0.008
Allergic rhinitis	6 (60.0)	6 (54.5)	2 (18.2)	0.118
Familial history of asthma	3 (30.0)	6 (54.5)	3 (27.3)	0.431
Current or former smoker	3 (27.3)	3 (27.3)	4 (36.4)	1.000
Severity of urticaria	13 (8, 19)	13 (4, 18)	11(1, 17)	0.280
IgE, IU/ml^†^	743.7 (539.7, 1580.4)	360.6 (218.2, 1197.7)	60.3 (35.2, 127.3)	< 0.001
The number of years after the diagnosis of CholU	0.6 (0.0, 8.1)	2.1 (0.0, 9.5)	0.6 (0.0, 4.3)	0.121
**Medication for CholU**				
Histamine H1 antagonist	9 (81.8)	10 (90.9)	3 (27.3)	0.003
Histamine H2 antagonists	6 (54.5)	9 (81.8)	1 (9.1)	0.003
Anti-leukotriene receptor antagonists	1 (9.1)	4 (36.4)	0 (0.0)	0.047

Values are shown as median (range) or n (%) unless otherwise indicated.

*CholU* cholinergic urticaria.

^†^Shown as median (25th, 75th percentile).

**Figure 2:**
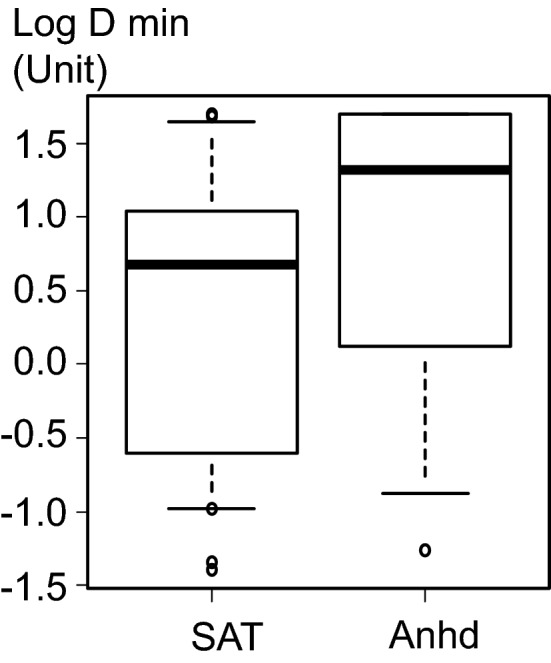
Log D_min_ between SAT (conventional sweat allergy-type CholU and CholU with palpebral angioedema) and CholU with acquired anhidrosis and/or hypohidrosis. *Anhd* acquired anhidrosis and/or hypohidrosis, *D*_*min*_ cumulative dose of inhaled methacholine when respiratory resistance began to increase, *SAT* sweat allergy type.

Table 2 shows the respiratory symptoms, FeNO level, and FEV1 (% predicted value) for the 3 groups. Respiratory symptoms evaluated with the IPAG questionnaire were less frequently observed in CholU-Anhd (0 [0, 1]) than in SAT-CholU (1 [0, 2]) or CholU-PA (1 [15]) (p = 0.049). FeNO of patients with SAT-CholU, CholU-PA, and CholU-Anhd was 23 (18.5, 65.0), 39 (32.0, 59.5), and 25 (19.0, 33.0) ppb, respectively. FeNO was elevated, although not significantly, in patients with CholU-PA compared to the other 2 subtypes (p = 0.237). Figure 2 shows log D_min_ between SAT (conventional SAT-CholU and CholU-PA) and CholU-Anhd. Log D_min_ tended to be lower in patients with SAT than in those with CholU-Anhd (median log D_min_, 0.676 and 1.318, respectively; p = 0.13). Median FeNO (25th, 75th percentile) was 35.5 (21.5, 64.8) in patients with SAT and 25 (19.0, 33.0) in those with CholU-Anhd (p = 0.175). FeNO was relatively low in patients with CholU-Anhd. Median FEV1 (% predicted) (25th, 75th percentile) was 87.4 (81.6, 89.9) in patients with SAT and 91.2 (80.5, 100.3) in those with CholU-Anhd (p = 0.294). One of the 2 patients with CholU-Anhd who had decreased FEV1 had a smoking history; meanwhile, 1 of 11 conventional SAT-CholU patients (9.1%) and 6 of 11 CholU-PA patients (54.5%) required treatment for asthma based on the decision of attending pulmonologist after physical examination and interview. There was no association between log D_min_ and severity of CholU symptoms (Spearman correlation coefficient = 0.21, p = 0.241).

**Table 2:** Respiratory symptoms, FeNO, and FEV1 (% predicted value) for the 3 subtypes of CholIU.

Variable	Conventional sweat allergy-type (n = 11)	CholU with palpebral angioedema (n = 11)	CholU with acquired anhidrosis (n = 11)	p value
IPAG questionnaire score	1 (0, 2)	1 (1, 3)	0 (0, 1)	0.049
FeNO	23 (18.5, 65.0)	39 (32.0, 59.5)	25 (19.0, 33.0)	0.237
FEV1 (% predicted)	87.2 (81.0, 89.4)	87.6 (81.8, 90.1)	91.2 (80.5, 100.3)	0.522

Values are shown as median (25th, 75th percentile).

*CholU* cholinergic urticarial, *FeNO* exhaled nitric oxide, *FEV1* forced expiratory volume in 1 s, *IPAG* international primary care airways group.

## Discussion

This is the first study investigating differences in bronchial hyperresponsiveness among subtypes of CholU. We showed that D_min_ was lower in patients with SAT (conventional SAT-CholU and CholU-PA) than in those with CholU-Anhd, although it did not differ significantly among the 3 subtypes. Respiratory symptoms were more frequently observed in patients with SAT and FeNO was elevated in patients with CholU-PA. Although the differences among the 3 subtypes were nonsignificant, this result may reflect the distinct pathogenesis of conventional SAT-CholU, CholU-PA, and CholU-Anhd. Namely, SAT is associated with sweat allergy and CholU-PA is closely related to atopic diseases^7^. A previous study investigating bronchial responsiveness in patients with CholU did not stratify the results based on CholU subtype^12^. Our study provides insight into the respiratory features of each CholU subtype based on a manifestation other than skin symptoms. Nine % of conventional SAT-CholU patients and more than half of CholU-PA patients required treatment for asthma based on the decision of attending pulmonologist after physical examination and interview. It is important to identify the subtype of CholU as this can determine the disease management strategy. A previous study showed that symptom duration and intensity were associated with bronchial hyperresponsiveness^12^, although we did not observe any association between the severity of urticaria symptoms and bronchial hyperresponsiveness. The study by Petalas et al. excluded patients with history of asthma, atopy, and smoking; under this study setting, the authors demonstrated that the respiratory symptoms of CholU resulted from bronchial hyperresponsiveness^12^; however, it is possible that they had fewer patients with SAT subtypes (conventional SAT-CholU and CholU-PA) than our study because they excluded patients with a history of atopic diseases. Not all of our patients with bronchial hyperresponsiveness required asthma treatment. We did not include normal subjects as a control group but in a previous report, log D_min_ was > 50 U in subjects with no history of asthma or other respiratory diseases and who had no current respiratory symptoms^14^. Bronchial hyperresponsiveness may be caused by CholU itself in some patients. In order to detect asthma, it is important to pay attention to respiratory symptoms, FeNO, and history of asthma as well as bronchial hyperresponsiveness. Our results also suggest that continuous methacholine inhalation is useful for evaluating bronchial responsiveness in CholU, which can reveal the underlying pathogenic mechanism in each subtype.

Our study had some limitations. Firstly, it was conducted at a single institution and had a limited sample size, which may have contributed to the lack of significant differences among the 3 CholU subtypes. Secondly, we did not exclude all confounding factors such as smoking history and history of asthma that can affect bronchial responsiveness. Although the proportion of smokers was similar across subtypes and there were no differences in baseline Rrs, these confounding factors may influence the result of bronchial responsiveness. Therefore, A multicenter study with a large sample size is needed to validate our findings. Additionally, future studies should address the prevalence of CholU in patients with asthma as a comorbidity.

In conclusion, log D_min_ tended to be lower in patients with SAT (conventional SAT-CholU and CholU-PA) than in those with CholU-Anhd. Distinguishing between subtypes of CholU may reveal different degrees of bronchial responsiveness based on differences in pathogenesis. And CholU-PA might be associated with asthma.

## Materials and methods

### Patients

Patients 16–80 years of age with CholU were prospectively enrolled. CholU was diagnosed and classified into subtypes by a dermatologist according to previously reported criteria^15^. Briefly, patients were diagnosed as cholinergic urticaria with provocation test such as exercise-induced test or acetylcholine intradermal injection. Sweat allergy was assessed by autologous sweat test. Patients who were contraindicated for methacholine inhalation challenge (e.g., severe airflow obstruction, recent asthma attack, or uncontrolled hypertension) or had uncontrolled asthma were excluded. The patients were divided into the following 3 groups: (1) conventional SAT-CholU, (2) CholU-PA, and (3) CholU-Anhd and follicular-type CholU. SAT was defined as (1) and (2), as both subtypes are associated with type I allergy to sweat.

This study was approved by the ethics committee of Kobe University (no. 160114) and was conducted in accordance with the Helsinki declaration. All patients provided written, informed consent before enrollment. If patients were under 20 years of age, their guardians also signed the agreement form. The study was registered with the University Medical Hospital Information Network of Japan (UMIN 000025669; https://upload.umin.ac.jp/cgi-open-bin/ctr/ctr_view.cgi?recptno=R000027550).

### Bronchial responsiveness

Bronchial responsiveness was evaluated by continuous methacholine inhalation challenge with simultaneous measurement of respiratory resistance (Rrs) using a previously developed device (Astograph; Chest, Tokyo, Japan)^14^. Patients inhaled twofold dilutions of methacholine chloride in saline (10 dose increments from 0.049 to 25 mg/ml) from nebulizers with an output of 0.15 ml of methacholine solution per min. Methacholine was inhaled at 1-min intervals until Rrs was > 2 times the baseline value or up to the maximum concentration of methacholine. The cumulative dose of inhaled methacholine when Rrs began to increase (D_min_) served as an indicator of bronchial responsiveness^14^. D_min_ was measured in units defined as inhalation of 1 mg/ml methacholine in 1 min. The total inhaled cumulative dose of methacholine at the highest dose (25 mg/ml) was 50 U. If there was no response and no elevation of Rrs even after inhalation of 50 U, a D_min_ of 50 was recorded, but this was considered as no bronchial responsiveness in the analyses. According to previous studies^14^, we analyzed D_min_ using the log10-transformed value, which has been validated in tests for bronchial responsiveness in clinical practice^16^. All participants stopped taking oral antihistamines, leukotriene receptor antagonists, theophylline, systemic, or inhaled corticosteroids, and inhaled long-acting β2 agonists for at least 72 h prior to assessment. The use of short-acting β2 agonists were permitted if participants have dyspnea from asthma exacerbation.

The pulmonary function test was performed using a spirometer (Auto Spirometer SYSTEM21; Minato Medical Science Co, Osaka, Japan). Exhaled nitric oxide (FeNO) was measured using an electrochemical NO analyzer (NIOX VERO; Aerocrine AB, Solna, Sweden).

Respiratory symptoms were assessed with the International Primary Care Airways Group (IPAG) questionnaire^17^. The clinical severity of CholU was assessed with the CholU severity index^18^, which is a summed score of symptom frequency (less than once a month = 0; once a month = 1; more than once a month = 2; once a week = 3; more than once a week = 4; daily = 5; and more than once daily = 6 points), eliciting factors (1 point each for physical exercise, hot bath, hot shower, emotional stress, hot food, sauna, and other), duration of skin lesions (< 5 min = 0; 5–10 min = 1; 10–20 min = 2; 20–30 min = 3; 30–60 min = 4; and > 1 h = 5 points), and itch (none = 0; mild = 1; moderate = 2; and severe = 3 points). The total score ranged from 0 to 21 points (< 5 points = very mild; 5–9 points = mild; 10–15 points = moderate; and > 15 points = severe).

### Endpoints

The study was designed as a prospective, single-center observational study. The primary endpoint was log D_min_ in the 3 subtypes of CholU, and the secondary endpoints were respiratory symptoms, FeNO, and forced expiratory volume in 1 s (FEV1, % predicted value). We also compared D_min_, respiratory symptoms, FeNO, and FEV1 (% predicted) between patients with SAT (conventional SAT-CholU and CholU-PA) and those with CholU-Anhd.

### Statistical analysis

Differences between groups were evaluated with the chi-squared test or Fisher’s exact test for qualitative data and the Kruskal–Wallis test for quantitative data. All statistical analyses were performed using EZR v1.38 (Saitama Medical Center, Jichi Medical University, Saitama, Japan), a graphical user interface for R v 3.3.2 software (R Foundation for Statistical Computing, Vienna, Austria)^19^.

### Ethics declarations

The study was performed in accordance with the Helsinki declaration and was approved by the ethics committee of Kobe University (No. 160114). And all the study’s participants signed a written informed consent.

## Supplementary Information

Supplementary Information.

## Data Availability

The datasets used and/or analysed during the current study are available from the corresponding author on reasonable request.
