# Supplementary material for: Cross-sectional study of cholinergic urticaria subtypes and bronchial hyperresponsiveness
Source: Sci Rep. 2022 Oct 27;12:18122. doi: 10.1038/s41598-022-22655-6 (PMC9613673; doi:10.1038/s41598-022-22655-6)

Cross-sectional study of cholinergic urticaria subtypes and bronchial hyperresponsiveness

Naoko Katsurada, Tatsuya Nagano, Masatsugu Yamamoto, Tatsunori Kiriu, Ryota Dokuni,

Hiroshi Kamiryo, Ai Yoshioka, Atsushi Fukunaga, Chikako Nishigori, Yoshihiro Nishimura,

Kazuyuki Kobayashi

Figure S1. Log2-transformed D_{min} of each subtype. Anhd, acquired anhidrosis and/or hypohidrosis; D_{min}, cumulative dose of inhaled methacholine when respiratory resistance began to increase; PA, palpebral angioedema; SAT, sweat allergy type.

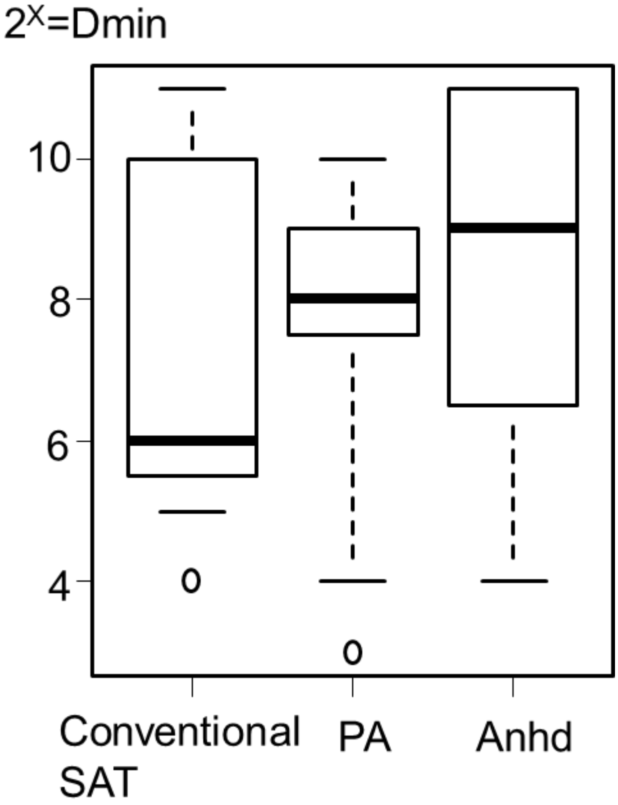

Supplement: Supplementary file 1 — Supplementary Information. [file 41598_2022_22655_MOESM1_ESM.pdf]
